# LEKTI-Grafted Sunflower Trypsin Inhibitor: A Potential Therapeutic for Skin Diseases

**DOI:** 10.1021/acs.jmedchem.5c01912

**Published:** 2025-11-11

**Authors:** Jeffrey Mah, Vignesh Jayarajan, Xin Huang, Wei-Li Di, Derek Macmillan

**Affiliations:** † Institute of Child Health, 4919 University College London, 30 Guilford Street, London WC1N 1EH, United Kingdom; ‡ Department of Chemistry, University College London, 20 Gordon Street, London WC1H 0AJ, United Kingdom

## Abstract

Kallikrein 5 (KLK5) is a serine protease expressed in the outer skin layers, where it regulates the barrier function by cleaving desmosomal proteins. Elevated KLK5 causes excessive proteolysis, leading to corneocyte overdesquamation and barrier compromise. Increased KLK5 activity has been linked to atopic dermatitis (AD), a chronic inflammatory disease affecting up to 20% of children, highlighting the need for therapies that restore barrier integrity. While many serine protease inhibitors have been developed, most lack KLK5 selectivity. To address this, novel analogues of the sunflower trypsin inhibitor were designed and evaluated. Lead **7** emerged as a potent (IC_50_ = 14 ± 4 nM; *K_i_* = 11 nM) selective KLK5 inhibitor. In keratinocytes from a Netherton syndrome patient, lead **7** significantly reduced the KLK5 activity and improved epithelial barrier integrity, as shown by transepithelial electrical resistance. These findings suggest lead **7** as a potential therapy for AD and other conditions with elevated KLK5 activity.

## Graphical abstract

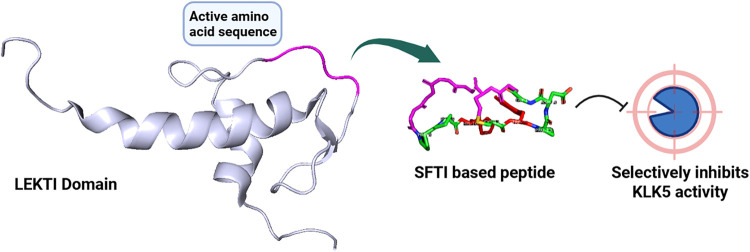

## Introduction

Kallikrein-related peptidase 5 (KLK5) is a serine protease expressed in the skin, particularly in keratinocytes.^1^ It plays a crucial role in epidermal homeostasis by regulating skin desquamation with other KLKs.^2,3^ KLK5 is able to autoactivate and initiate a proteolytic cascade by activating other kallikreins, notably KLK7 and KLK14.^4^ KLK5-mediated cleavage of desmoglein-1 (DSG1) has been demonstrated in studies on Netherton syndrome (NS), where enhanced activated KLK5, due to loss of function of the endogenous serine protease inhibitor lympho-epithelial Kazal-type–related inhibitor (LEKTI), underlies this disease.^5^ Studies suggest that high KLK5 activity contributes to excessive corneocyte shedding, increased trans-epidermal water loss (TEWL), and barrier dysfunction.^6,7^

Elevated KLK5 activity, mRNA, and protein expression have been reported in skin with AD.^8,9^ It is considered that enhanced KLK5 activity in AD might contribute to barrier dysfunction as KLK5 degrades corneodesomal proteins such as DSG1, resulting in abnormal desquamation and impairment of skin barrier integrity.^8–12^ Polymorphisms in the *SPINK5* gene, which encodes the LEKTI protein, may lead to enhanced KLK5 activity in atopic dermatitis (AD).^10,13^ Skin barrier dysfunction, along with immune-response dysregulation, is strongly associated with pathogenesis in AD.^14–16^ The skin barrier plays a crucial role as a physical and functional barrier, protecting the body from environmental irritants, allergens, and microbial invasion. However, current therapies for AD mainly target immune-response dysregulation and not the underlying causes of barrier dysfunction, for example, enhanced KLK5. Controlling KLK5 activity and restoring skin barrier integrity can, therefore, be a specific therapeutic strategy for AD patients.^17^

An ideal serine protease inhibitor should exhibit both high potency and specificity. High potency allows for the use of lower therapeutic doses, minimizing unintended off-target interactions. Selective inhibition is also important, as multiple kallikreins, including KLK7 and KLK14, are expressed in the skin with distinct physiological functions. For example, KLK7 controls corneocyte detachment by degrading corneodesmosomes such as corneodesmosin (CDSN), DSG1, and desmocollin-1 (DSC1),^7^ while KLK14 modulates extracellular matrix (ECM) remodelling by breaking down fibronectin and laminin.^18,19^ Broad inhibition of several kallikreins risks disrupting these essential processes, leading to unwanted physiological effects. By contrast, focusing specifically on KLK5 offers several advantages: it is more straightforward to monitor the biochemical and cellular consequences of selective inhibition, limits the risk of cross-reactivity and off-target activity, and allows for a clearer definition of downstream molecular pathways and dose-dependent efficacy and toxicity. At present, there is no universally recognized standard for the selectivity window of kallikrein inhibitors. In dermatological applications, however, achieving selectivity for KLK5 of several hundred to several thousand-fold is considered meaningful. This benchmark applies both in comparison with other skin-expressed kallikreins, such as KLK7 and KLK14, and with other trypsin-like serine proteases. In addition, topical administration of a KLK5 inhibitor is expected to mitigate potential concerns by restricting the pharmacological activity to the skin surface. A potent and selective KLK5 inhibitor is therefore required to achieve therapeutic efficacy with improved safety, and several efforts have been directed toward its development. For example, small molecule inhibitors such as chloromethyl ketone and GSK 951 covalently bind to KLK5 at its catalytic serine;^17,20^ peptide inhibitors, particularly cyclic peptides derived from sunflower trypsin inhibitor-1 (SFTI-1) and its analogues;^21^ as well as endogenous inhibitors such as LEKTI.^22,23^

Kallikreins possess a substrate-binding pocket whose specificity varies among family members, enabling some to discriminate effectively between trypsin-like and chymotrypsin-like substrates.^24^ In fact, the specificity of each KLK arises from additional interactions over a large surface involving multiple amino acid sites. Small molecules struggle to engage multiple amino acid sites, while large proteins can provide a large interaction surface but face delivery and stability challenges. Cyclic peptides serve as an effective bridge between small molecules and larger proteins, offering a promising alternative for drug development.

Sunflower trypsin inhibitor-1 (SFTI-1) **1** is a cyclic peptide comprising 14 amino acids, produced naturally from sunflower seeds, and demonstrates potent inhibition of trypsin.^21,25^ This peptide serves as an excellent structural scaffold for the engineering of protease inhibitors due to its exceptional stability and specificity. One common method to enhance its efficacy involves screening point mutations to discover variants that show heightened activity against specific proteases.^25–27^ Additionally, SFTI-based libraries have been deployed to identify effective inhibitors across a spectrum of targets.^27–29^ Our team has developed various analogues, including **2**, a triple mutant with the amino acid sequence *cyclo*-[GRCT**R**SIPP**H**C**W**PD] (Figure 1A). **2** showed enhanced specificity and binding affinity toward KLK5 (IC_50_ 56 ± 0.5 nM) and KLK14 compared to **1**. **2** includes three key substitutions composed of histidine for isoleucine at position 10 (I10H), arginine for lysine at position 5 (K5R), and tryptophan for phenylalanine at position 12 (F12W) (Figure 1A). However, although **2** showed an increased inhibition on KLK5 relative to **1**, it displayed dual inhibition of both KLK5 and KLK14.

**Figure 1:**
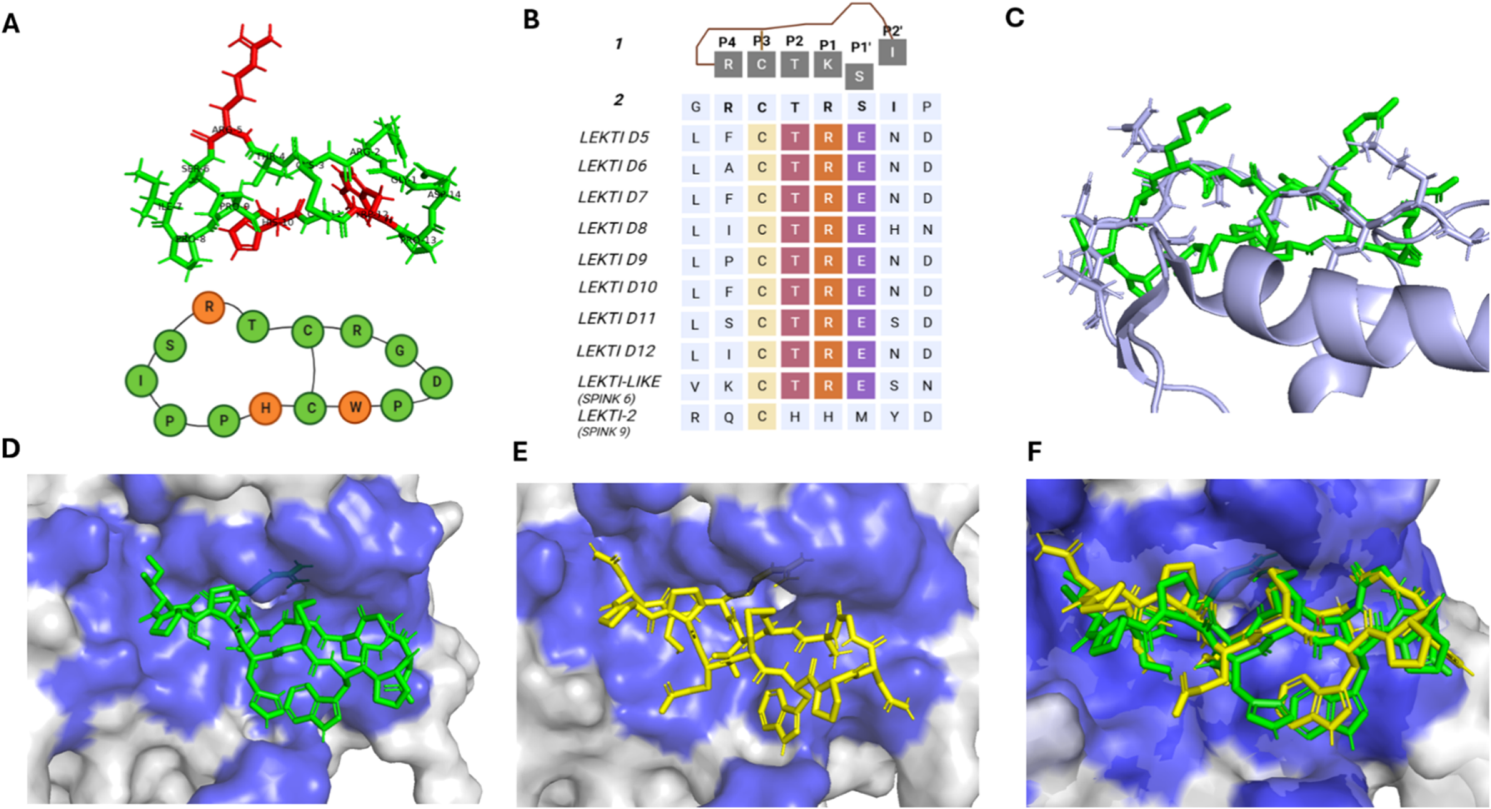
(A) SFTI-1 (**1**) served as the template from which **2** was derived, as displayed, highlighting substitutions (red) in amino acid residues. (B) Sequence alignment of **1** and **2** with LEKTI domains is shown, emphasizing conserved and variable regions. (C) A structural overlay of the LEKTI domain (gray ribbon; PDB: 1H0Z) and 2 (green sticks; derived from PDB: 4XOJ) illustrates the conservation of backbone structure and modifications for side chains. (D) The docked complex of **2** (green sticks; derived from PDB: 4XOJ) within the binding pocket of KLK5 (blue surface; PDB: 2PSX), with an RMSD of 1.2 Å compared to defined binding backbone atoms of the flexible interface for both KLK5 (Ser195, His57, and Asp102) and **2**, binding free energy (Δ*G*) of −10.4 kcal/mol, and a dissociation constant (*K*_d_) of 2.4e-08 M. (E) The predicted docking configuration of KLK5 (blue surface; PDB: 2PSX) with an SFTI variant grafted with LEKTI domain 6 (yellow sticks; derived from PDB: 4XOJ), shows an RMSD of 0.2 Å, Δ*G* of −10.1 kcal/mol, and *K*_d_ of 3.7e-08 M. (F) An overlay of the docking poses of **2** (green sticks) and grafted SFTI variant (yellow sticks; derived from PDB: 4XOJ) within the KLK5 binding pocket (blue surface; PDB: 2PSX) is depicted, comparing their binding conformations.

To address the issue of specificity, researchers have applied a “grafting” strategy to the SFTI-1 scaffold. Instead of replacing a single amino acid, partial or whole bioactive sequences from an endogenous protein are selected and grafted onto the SFTI-1 scaffold.^30^ It has been reported that grafted SFTI-1 analogues possessed biological functions similar to full-length proteins while avoiding undesirable interactions.^31–35^ The first study, which grafted active amino acid sequences from Bowman-Birk inhibitor (BBI) onto the SFTI-1 cyclic backbone for trypsin inhibition, was performed in 2004.^36^ This graft produced an SFTI analogue that is symmetrical and functions as a bifunctional inhibitor, while retaining structural rigidity.^36,37^ Subsequently, SFTI analogues grafted with active amino acid sequences from kallistatin, α_1_-antichymotrypsin, and protein C inhibitor were also reported, and these analogous were able to inhibit KLK7 with a *K_i_* of 0.4, 0.5, and 0.7 μM respectively.^38^

**2**, as a small peptide, possesses rare and valuable properties, making it a powerful tool for drug design. These qualities enable its use as a molecular grafting scaffold for targeting various therapeutic classes beyond proteases. Our strategy to develop potent and specific peptide inhibitors, based on **2**, began with computational modeling referencing the endogenous inhibitor LEKTI to predict KLK5 binding interactions. These models led to the design of peptides that effectively bind and inhibit KLK5, preventing the proteolytic cascade responsible for skin damage. This approach offers a highly selective and potent therapeutic option, potentially restoring proteolytic balance and alleviating KLK5-related dermatological diseases. It could provide treatments toward a specific molecular dysfunction in skin disorders.

## Results and Discussion

### Computational Study

KLK5, a serine protease with trypsin-like activity, presents a significant challenge for selective inhibitor development due to its conserved catalytic domain and a 45.9% sequence similarity with trypsin. To address this challenge, researchers have explored natural inhibitors, particularly LEKTI, which is encoded by the *SPINK5* gene and exhibits strong KLK5 inhibition. Like SFTI-1 and similar inhibitors, LEKTI’s P1 residue primarily determines specificity for trypsin or chymotrypsin, with adjacent residues P2 and P3 also contributing to binding efficacy. Analysis of individual LEKTI domains with known binding interactions toward serine proteases revealed limited variation in two of its 12 contact residues—P3 (a conserved cysteine) and P15′ (a conserved asparagine), while the other ten residues exhibited high variability. Notably, the sequence of LEKTI’s residues Cys (P3), Thr (P2), and Arg (P1) closely matches those of SFTI-1 (Figure 1B). Structural analysis of available LEKTI domains—specifically Domain 4 (RCSB: 5YHN), Domain 6 (RCSB: 1H0Z), and the LEKTI-like *SPINK6* (RCSB: 2N52)—revealed backbone similarities with SFTI-1, particularly in their inhibitory loops, suggesting feasible interactions with KLK5 (Figure 1C). To further evaluate this potential, the KLK5 crystal structure (PDB: 2PSX) and a modified SFTI-1 solution structure (PDB: 1JBL) were used in docking studies employing high-ambiguity-driven protein–protein docking (HADDOCK) (Figure 1D). Additional docking simulations incorporating LEKTI Domain 6 (Figure 1E) yielded binding interactions and stability comparable to those of the initial SFTI-KLK5 interactions (Figure 1F).

From docking, the KLK5 enzyme’s subsites (S4–S1) exhibit distinct characteristics influencing interactions with SFTI’s P4–P1 residues. Docking analysis reveals that the open, hydrophobic S4 subsite shows minimal specificity but slightly favors hydrophobic P4 residues. The S3 subsite is less selective, with P3 side chains extending into the solvent, impacting stability but not specificity. Notably, **2** requires a cysteine at P3 for a disulfide bond, limiting optimization. The S2 subsite forms a wedge-shaped, polar pocket due to His99 and His57, accommodating the P2 side chain. However, specificity is primarily dictated by the S1 pocket, which facilitates trypsin-like recognition. The P1-Arg side chain deeply inserts into S1, forming a stabilizing salt bridge with Asp189’s carboxylate, along with hydrogen bonds to Ser190, a water molecule, and Asp217’s carbonyl.

LEKTI comprises 15 domains, several of which have been identified as potent KLK5 inhibitors. However, limited research has been conducted on the inhibitory properties of the individual domains. Individual and promising domains that have the greatest potential as KLK5 inhibitors were prioritized. The majority of the LEKTI domains feature positively charged arginine at the P1 position, which is particularly favorable for trypsin-like serine proteases such as KLK5. Computational analysis and prior findings identified LEKTI Domains 8–11 as potent KLK5 inhibitors, with Domains 5 and 6 exhibiting high inhibitory activity (*K_i_* values of 32.8 and 83.3 nM, respectively). Furthermore, Domain 10 was selected due to its conserved binding site within the LEKTI domains, sharing the same sequence with domains 7 and 5. LEKTI-2, encoded by *SPINK9*, showed selective binding to KLK5 (*K_i_* = 65 nM) without potently inhibiting KLK7 or KLK14. LEKTI-2 (*SPINK9*) features a histidine at P1, which was identified as suboptimal for KLK5′s S1 pocket due to its bulkiness. Substituting histidine with arginine, known for its positively charged side chain and compatibility with trypsin-like enzymes, was proposed to enhance the binding efficiency. Therefore, LEKTI domains 10 and LEKTI-2 were selected for further investigation and grafting potential.

### Chemistry

Building on the improved potency and selectivity of **2**, a strategy was employed to graft key residues (P1, P2, P3, P4, and P2′) from LEKTI domains onto **2** to enhance the KLK5 potency and selectivity. This approach aimed to convert a large protein-based inhibitor into a smaller, more stable peptide inhibitor with simpler synthesis and delivery (Figure 2A). Optimized LEKTI-derived sequences were prioritized, leading to the creation of four new analogues targeting KLK5. The linear **2**, now integrated with LEKTI and LEKTI-like domains for KLK5 inhibition studies, was synthesized using solid-phase peptide synthesis, achieving isolated yields between 10 and 30%. Subsequent steps included native chemical ligation for cyclization, followed by oxidation to produce the final products (Figure 2B). These were verified using analytical HPLC for purity assessment, achieving a purity above 95% (Figure 2C) and LC-MS to confirm peptide identity (Figure 2D, see Supporting Information for characterization of all KLK5 inhibitor candidates).

**Figure 2:**
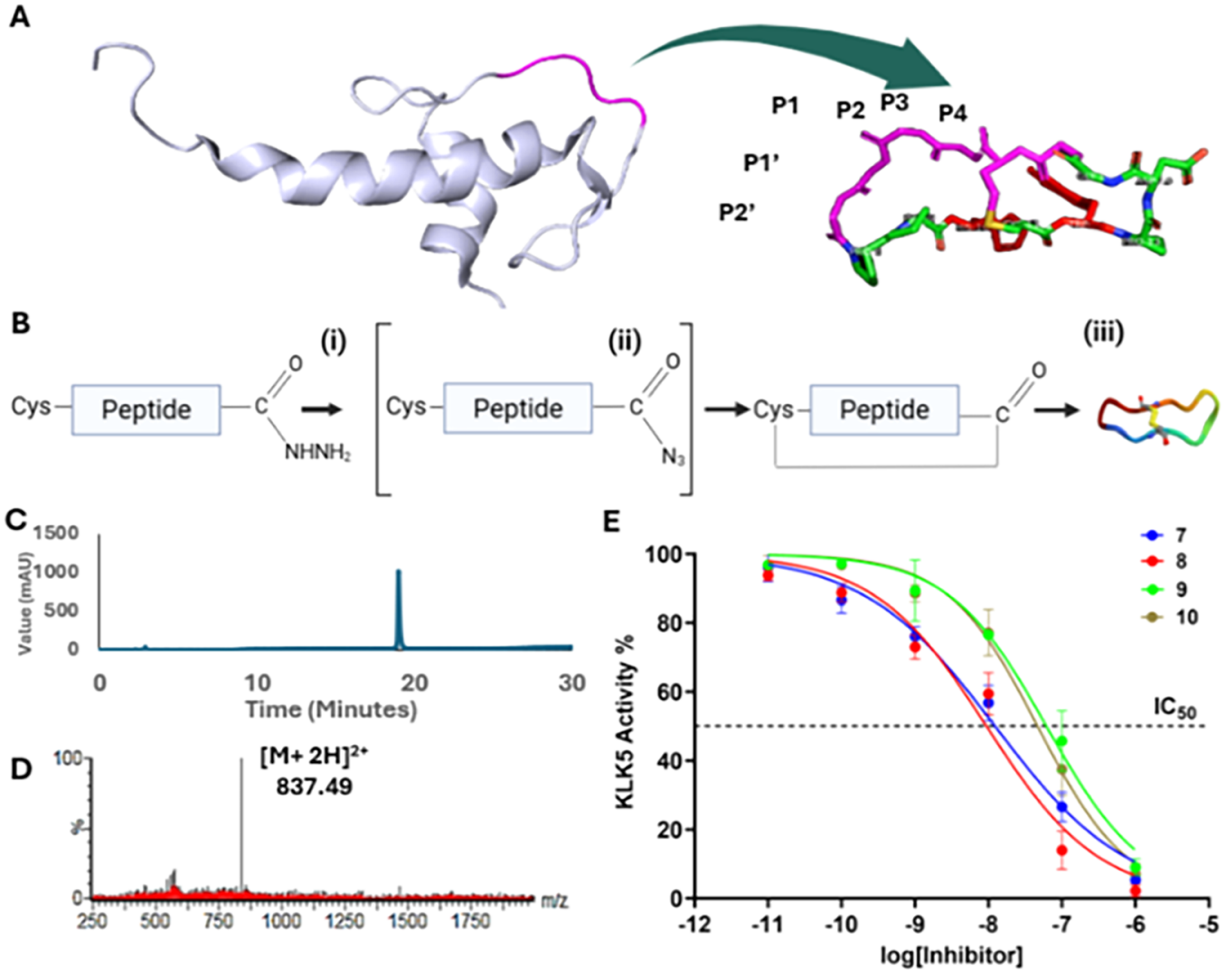
(A) Process of grafting key residues of LEKTI domains onto SFTI is illustrated, showing the peptide transformation. (B) The peptide synthesis process is outlined in a schematic that includes the following steps: (i) activation using NaNO_2_ (10 equiv) in a 0.2 M phosphate buffer containing 6 M Gn-HCl at pH 3.0, −15 °C for 15–20 min, (ii) native chemical ligation with MPAA (40 equiv) in 0.2 M phosphate buffer at pH 7 conducted over 4 h, and (iii) air oxidation at pH 8.0 in a 50 mM ammonium bicarbonate solution for 24 h. (C) Analytical HPLC results display over 95% purity for the synthetic grafted peptide. (D) Mass spectrometry data confirms the correct mass of the peptide: *m*/*z* = 837.49 Da for 2+ ion (MW = 1673 Da) for **7**. (E) IC_50_ data for the grafted SFTI analogues are shown in a plot, indicating the inhibitory concentrations against KLK5, with *n* = 3.

Inhibitory activity against KLK5 was evaluated using the fluorogenic substrate Boc-VPR-AMC for KLK5 (Table 1). Initial grafting resulted in a compromised inhibitor, likely due to serine-to-glutamic acid substitution within the LEKTI domain, shifting IC_50_ values into the micromolar range (**3**: 1799 ± 58 nM; **4**: 1492 ± 50 nM). The P1–P1′ reactive site (Arg5 to Ser6) is critical for inhibitor binding, with the P1′ position contributing up to 50% of the total association energy.^39,40^ The conserved serine at P1′ in Bowman-Birk inhibitors (BBIs) is essential for function. While Ser6 is not strictly required, substituting it with alanine in SFTI-1 reduces binding affinity significantly, increasing the *K*_d_ by 11-fold,^41^ highlighting the importance of its hydroxyl group in stabilizing interactions as evident by compromised inhibitory activity against KLK5 for **3** and **4**. Preservation of serine in the P1′ position restored inhibitory activity seen in **5** and **6**. Preserving the native serine restored efficacy to the nanomolar range (**5**: 31 ±  7 nM; **6**: 114  ±  11 nM).

**Table 1:** Summary Table of SFTI Analogues

		IC_50_ (*n* = 3)/nM for KLKs			
**#**	sequence^a^	KLK5	KLK7	KLK8	KLK14
**1**	DFCTKSIPPICFPD	230 ± 3^b^		1286 ± 51^b^	30 ± 7^b^
**2**	GRCT**R**SIPP**H**C**W**PD	54 ± 3	534^b^	373 ± 31^b^	24 ± 2^b^
**3**	G**FCTREN**PP**H**CWPN	1799 ± 58			
**4**	G**FCHREY**PP**H**CWPN	1492 ± 50			
**5**	G**FCTR**S**N**PP**H**CWPN	31 ± 7			
**6**	G**FCHR**S**Y**PP**H**CWPN	114 ± 11			
**7**	G**FCTR**S**N**PP**E**CWPN	14 ± 4	>1 μm	>1 μm	>1 μm
**8**	G**FCHR**S**Y**PP**E**CWPN	8.6 ± 2	>1 μm	>1 μm	170
**9**	G**SCTR**S**S**PP**E**CWPN	54 ± 6	165 ± 12	>1 μm	>1 μm
**10**	G**YCTR**S**S**PP**E**CWPN	56 ± 7	240 ± 14	>1 μm	>1 μm

a Substituted amino acids are highlighted in bold.

b Data derived from Chen et al. (2016).

In our previous studies, N-to-S acyl shift chemistry required a P5′ I10H substitution for **2–6**, which proved to be suboptimal for KLK5 binding. As KLK5 favors acidic residues at P5′, particularly glutamic acid that engages Lys60 through electrostatic interaction, histidine was replaced with glutamic acid to optimize the graft design for **7–10**. Further backbone modifications on position 10 with glutamic acid enhanced potency. Optimized SFTI grafts, including **7** and **8**, showed markedly enhanced inhibitory activity. Successful grafting of additional domains, such as **9** and **10**, further demonstrated the strategy’s broader applicability (Figure 2E). Analyses showed that the grafted SFTI analogues exhibited a significant increase in the inhibitory efficacy (Figure 2E). Notably, **7** demonstrated an IC_50_ of 14 ± 4 nM (*K_i_* = 11 nM), marking a 4-fold increase compared to **2** (IC_50_ = 54 ± 3 nM), and a 16-fold increase relative to **1** (IC_50_ = 230 ± 3 nM). Among the tested analogues, **8** emerged as the most potent, with an IC_50_ of 8.6 nM ± 2 (*K_i_* = 8 nM), a 7-fold enhancement compared to **2** and a 28-fold increase over **1**. This suggests that the specific grafting strategies, particularly those for **8**, markedly improved binding and inhibition properties. **9** and **10** displayed comparable inhibitory activity to **2**. The results indicated that beneficial amino acids from LEKTI could be effectively incorporated into the SFTI scaffold without diminishing the inhibitory function.

The interaction between conserved Ser6 and Thr4 is vital for properly orienting the P1-Arg side chain for binding to the enzyme’s S1 pocket. Analogues with polar P4 substitutions, such as serine and tyrosine (e.g., **9** and **10**), exhibited comparable potency to **2**, likely due to favorable polar interactions with KLK5′s Gln-175 side chain in the S4 subsite. In contrast, **7** and **8**, with nonpolar P4 residues, showed improved KLK5 inhibition despite not having a favorable residue for KLK5 interactions, suggesting the mechanism may involve enhanced intramolecular stability rather than direct binding interactions. Further optimization of these interactions could enhance KLK5 inhibitor potency, emphasizing the importance of structural positioning and binding kinetics. **8**, featuring an aromatic residue at P2′, showed the most effective KLK5 inhibition, likely due to π-oriented interactions with Phe150 in the S2′ pocket. Other analogues, lacking aromatic residues at P2′, still retained inhibitory activity, suggesting that other residues and intermolecular interactions contribute to potency.

In a clinical context, several trypsin inhibitors are approved and used therapeutically, providing a benchmark for assessing the potency. Aprotinin, employed to reduce perioperative bleeding, inhibits trypsin-like plasmin with a *K_i_* of 0.07 nM.^42^ Gabexate mesylate, approved for acute pancreatitis and coagulation disorders, inhibits trypsin, plasmin, plasma kallikrein and thrombin (IC_50_ values are 9.4, 30, 41, and 110 μM respectively).^43^ Taken together, these data indicate that lead **7** fits well within the clinical context of trypsin inhibitor potency, warranting further assessment of its selectivity among trypsin-like proteases. In terms of selectivity, major KLKs found in the epidermal layer of the skin were assessed for cross-reaction. KLK7 was included because of its prominent distribution in both the basal and upper layers of the epidermis,^12^ KLK8 because of its presence in the stratum corneum, stratum granulosum, and skin appendages, and KLK14 because it is the most skin-specific KLK with relatively high abundance compared to other tissues.^19,44,45^ **9** and **10** displayed inhibitory properties toward KLK7. Despite **9** and **10** being less potent for KLK5, they still offer 4-fold selectivity against select KLKs in skin. In the case of compound **8**, KLK14 was inhibited despite being potent against KLK5. However, it was excluded from further optimization due to cross-reactivity with KLK14. On the other hand, **7** does not interact with any KLKs found in the skin, suggesting a potent and selective candidate for further investigation.

### Biological Evaluation

PAR2, a member of the G-protein-coupled receptor family expressed in keratinocytes, is a known downstream target of KLK5. KLK5 activates PAR2 by cleaving its extracellular N-terminal domain. This cleavage exposes a tethered ligand, which binds PAR2 intramolecularly to activate the receptor.^46,47^ Activated PAR2 causes protein kinase C (PKC) and inositol 1,4,5-trisphosphate (IP3) activation, leading to calcium release from the endoplasmic reticulum, known as intracellular calcium mobilization. To determine whether the lead **7** could inhibit KLK5-mediated PAR2 activation, a PAR2-dependent calcium mobilization assay was performed in an immortalized keratinocyte cell line (N/TERT).^48,49^ Following stimulation using a PAR2 agonist peptide (AP) in keratinocytes, PAR2-dependent calcium mobilization was observed (Figure 3A, blue curve), while following the addition of recombinant KLK5 (rKLK5), a calcium mobilization with a slight delay was observed (Figure 3A, green curve). This delay is attributed to the time required for KLK5 to enzymatically cleave the extracellular N-terminal domain of PAR2, thereby exposing the tethered ligand sequence necessary to initiate receptor activation and downstream calcium signaling. The peptide with reversed AP sequences (R-AP) was used as control, and the results showed that it did not induce calcium mobilization (Figure 3A, red curve). This observation confirmed PAR2-dependent calcium mobilization and that rKLK5 could initiate PAR2 activation. The inhibition potential of lead **7** on KLK5 activity was then assessed by using this assay. rKLK5 was preincubated with lead **7** at concentrations of 2.5, 5, 20, or 40 μM for one hour before being applied to cultured keratinocytes, after which PAR2-dependent calcium mobilization was measured. Results showed a dose-dependent reduction in PAR2-dependent calcium mobilization (Figure 3B). A complete inhibition of calcium mobilization was achieved at the concentration of 10 μM of lead **7**, which was an approximate 1:1 molar ratio of the inhibitor to rKLK5. This result demonstrated that lead **7** at a concentration of 10 μM could effectively inhibit KLK5.

**Figure 3:**
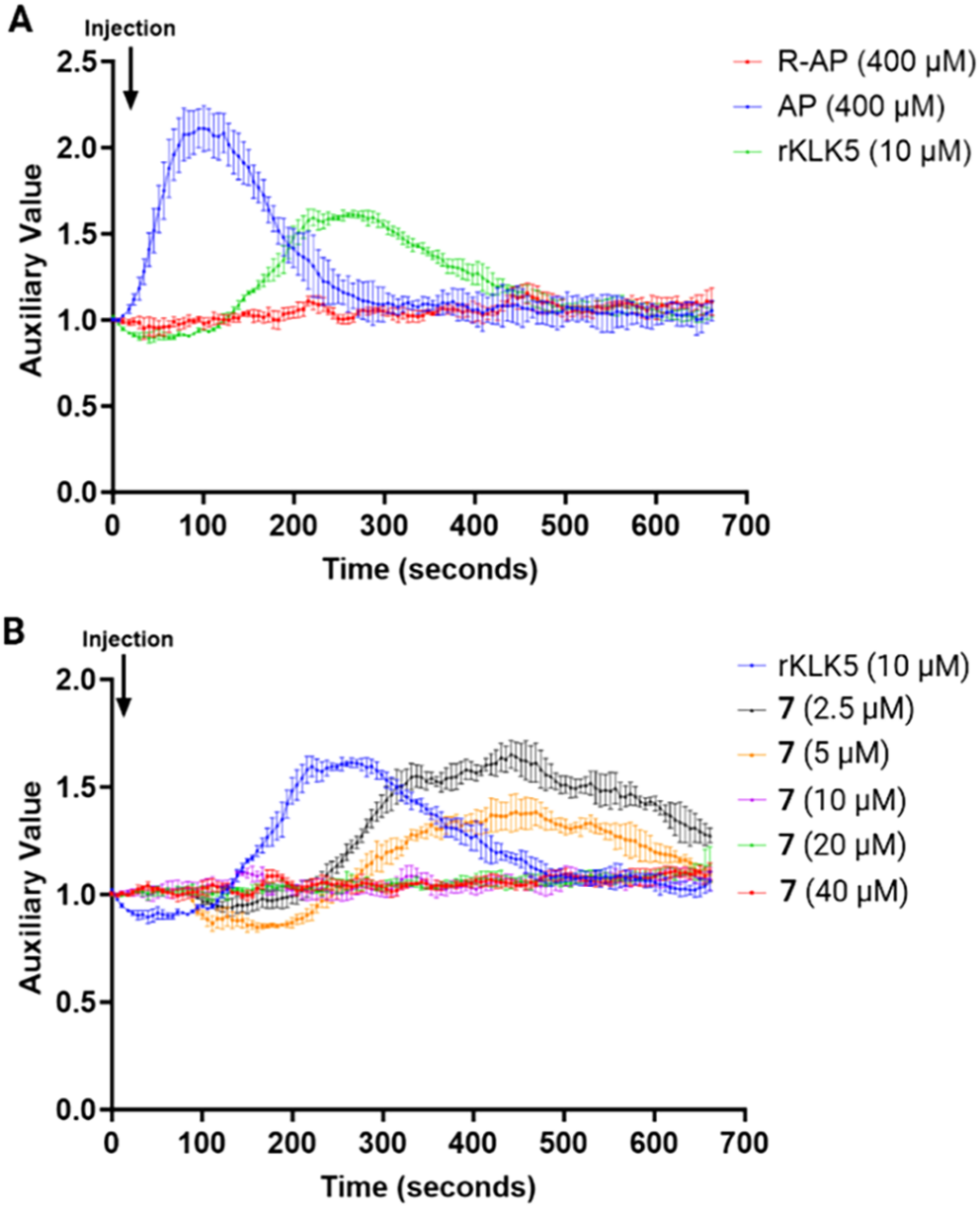
Calcium signaling dynamics in response to KLK5 and inhibitor treatments. (A) Time-course analysis of calcium signaling in keratinocytes following stimulation with recombinant KLK5 (rKLK5, 10 μM), agonist peptide (AP, 400 μM), and reversed sequence agonist peptide (R-AP, 400 μM). (B) Dose-dependent calcium signaling response to increasing concentrations of lead **7** (2.5 μM to 40 μM) in keratinocytes, demonstrating a progressive reduction in calcium release (*n* = 3).

Netherton Syndrome (NS), known for its elevated KLK5 activity, was used as a disease cell model. To determine whether NS keratinocytes have increased KLK5, KLK5 activity was measured in NS keratinocytes and compared to that of keratinocytes from three healthy donors (*n* = 3). Keratinocytes were seeded in 6-well plates and cultured to confluence to allow keratinocyte differentiation and KLK5 secretion. Upon reaching confluence, cultures were maintained in serum-free medium for an additional 48 h. Media was then collected and precipitated for KLK5 activation measurement using the protease activity assay.^50^ In NS keratinocytes, KLK5 activity was 51.9 ±  1.6 nmol/g (*n* = 3), whereas in healthy donor keratinocytes, KLK5 activities averaged 11.9 ±  0.6 nmol/g, with a range of 9 to 15 nmol/g (*n* = 9). While baseline protease levels can differ from one donor to the next, this confirmed a higher KLK5 activity in NS keratinocytes than that in healthy donors.

To evaluate the inhibition of lead **7** on endogenous KLK5 activity, media collected from cultured NS keratinocytes and healthy donor keratinocytes were incubated with lead **7** at the final concentration of 100 nM, or 1 μM, or 10 μM for one hour. Media were then concentrated, and KLK5 activity was measured. The results showed KLK5 activity in NS keratinocytes following lead **7** treatments were 44.3 ± 2.7 nmol/g for 100 nM, 33.5 ± 2.6 nmol/g for 1 μM, and 30.5 ± 1.7 nmol/g for 10 μM. There were significant differences in KLK5 activity before and after treatment (*p* < 0.05, *n* = 3). The reduction in KLK5 activities at each concentration was 14.6, 35.5, and 41.2%, respectively (Figure 4A). In normal donor keratinocytes, there were no significant differences before and after treatment (*p* > 0.05, *n* = 9). These results showed that lead **7** was able to inhibit enhanced endogenous KLK5 in keratinocytes but had a minimal effect in healthy donor keratinocytes. This may be because lead **7** exerts inhibitory effects only when KLK5 activity exceeds a certain threshold, which is not reached in keratinocytes from healthy donors. It was noted that the activity of KLK5 in NS was not reduced to the levels observed in normal keratinocytes. Two reasons might be behind this: (i) a higher lead **7** dose may be required, and (ii) the VPR-AMC substrate can be cleaved by a wider range of serine proteases. The remaining noninhibitory activity could be attributed to the presence of other trypsin-like serine proteases secreted in the cultured medium, which can hydrolyze the VPR-AMC substrate but are not inhibited by lead **7**.

**Figure 4:**
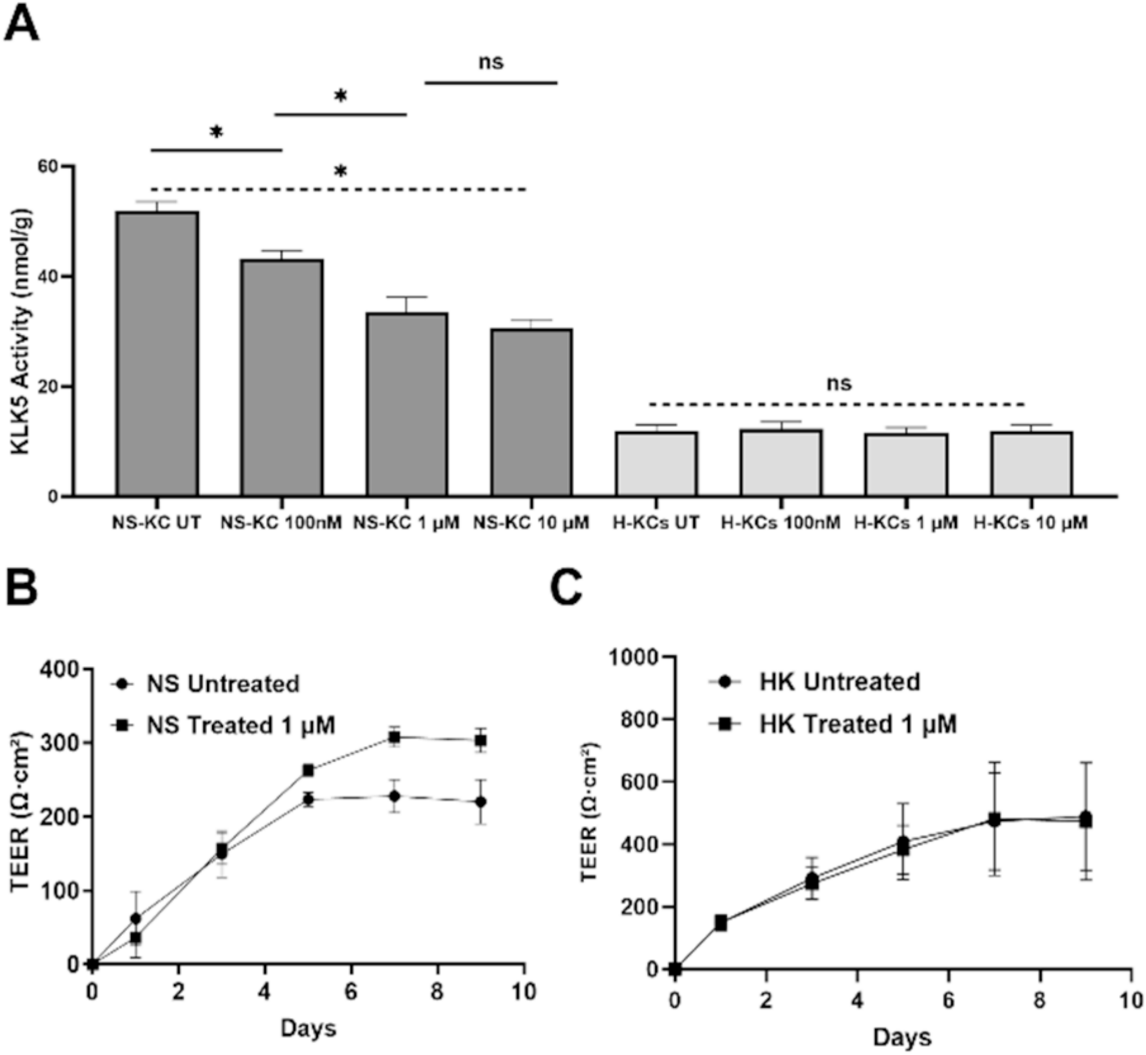
KLK5 activity in keratinocyte cell lines and its impact on cell barrier function. (A) KLK5 activity was measured in culture media from NS keratinocytes and healthy donor keratinocytes treated with varying concentrations of lead **7**, by a protease activity assay. Bars represent mean KLK5 activity levels (nmol/g) for one NS keratinocyte cell line and three healthy donor keratinocytes. The experiment was repeated three times. * Represents *p* < 0.05; N.S represents no significance with *p* > 0.05. (B) TEER was measured over 9 days in a single NS keratinocyte line. (C) TEER measurements over 9 days for pooled samples from three healthy donor keratinocyte lines.

KLK5 regulates epidermal barrier homeostasis by targeting corneodesmosomal proteins involved in cell–cell adhesion, including desmoglein-1 (DSG1), desmocollin-1 (DSC1), and corneodesmosin (CDSN).^7^ Dysregulated KLK5 activity can accelerate the degradation of these adhesion molecules, leading to impaired corneocyte cohesion and disruption of the epithelial barrier. To assess whether control of KLK5 activity by lead **7** could improve barrier integrity, the resistance of epidermal sheets generated using NS and healthy donor keratinocytes was measured using a transepithelial electrical resistance (TEER) assay. Keratinocytes from NS keratinocyte and three healthy donors were seeded in 6-well plates and cultured in the medium with 1 μM of lead **7** or without. The medium was replaced every 3 days with fresh lead **7** added in the treated groups. TEER measurements were performed every other day, and cultures were maintained until TEER values reached a plateau (9 days). In NS keratinocytes, there was a significant difference between treated and untreated, with an obvious increase in TEER values when keratinocytes reached confluence to form an epithelial sheet (*p* < 0.05, *n* = 18; Figure 4B). In contrast, there were no differences in TEER reading between treated and untreated groups in healthy donor keratinocytes (*p* > 0.05 *n* = 54; Figure 4C). These results suggested that lead **7** could improve barrier integrity by the inhibition of KLK5.

## Conclusions

The grafting of specific LEKTI-derived residues onto the SFTI scaffold significantly enhanced inhibitory potency and selectivity, with lead **7** exhibiting an IC_50_ of 14 ± 4 nM (*K_i_* = 11 nM) against KLK5 and little cross-reactivity with other KLKs. Functional studies in keratinocytes confirmed that lead **7** suppressed KLK5-mediated activation of PAR2. Moreover, in an NS disease cell model, lead **7** markedly reduced KLK5 activity in a dose-dependent manner and improved epithelial barrier integrity. This study demonstrated that the grafted SFTI-based compound lead **7** is an effective and selective inhibitor of KLK5. Further optimization and in vivo tests are necessary to address how to deliver the peptide into the skin.

## Experimental Section

### 1 General Procedures

All solvents and reagents were purchased from Sigma-Aldrich (U.K.) and used without further purification. Preparative reversed-phase high-performance liquid chromatography (RP-HPLC) was carried out on a Dionex Ultimate 3000 system with a Phenomenex Jupiter 10 μm Proteo 90 Å, C_12_ column (250 mm × 21.2 mm), using a gradient of 5–60% acetonitrile in water (both with 0.1% TFA, v/v) and monitored at 230, 254, and 280 nm. Analytical RP-HPLC employed a Phenomenex SphereClone 5 μm ODS, C_18_ column (250 mm × 4.6 mm), with a gradient of 5–95% acetonitrile (0.1% TFA) and identical detection wavelengths. All compounds are >95% pure by HPLC analysis. Analytical LC-MS was performed using a Waters Acquity UPLC SQD with a BEH C_18_ column (2.1 mm × 50 mm) eluted with a 5–95% gradient of acetonitrile (0.1% formic acid) in water at 254 nm. Ultraperformance LC-MS was also conducted using a Waters SQD2 system and Thermo Scientific C_4_ column (1.9 μm, 2.1 mm × 150 mm) at a flow rate of 0.6 mL/min, with detection at 254 nm.

### 2 Synthesis of Linear Peptides (Analogues **2–6**)

Analogues **2–6** were synthesized manually via Fast-Moc solid-phase synthesis using preloaded Fmoc-Cys­(Wang) resin (0.7 mmol/g, 0.05 mmol scale). First, Fmoc deprotection was carried out with 20% piperidine in DMF (1 mL, 20 min) and the resin was washed (3× DMF, 3× DCM). Amino acids were preactivated by mixing 10 equiv, dissolved in DMF (1.1 mL), with 10 equiv of HBTU/HOBt (1.1 mL from a 0.45 M stock in DMF) and 17.5 equiv of DIPEA (150 μL) in a 15 mL glass vial with stirring. This solution was transferred to the resin-containing reaction vessel, and coupling was performed for at least 1 h with agitation at 430 rpm. The reaction vessel was drained, and Fmoc deprotection was carried out as before. Following peptide chain assembly, the resin was washed (3× DMF, 3× DCM), and the peptides were cleaved with TFA/EDT/H_2_O (95:2.5:2.5, v/v/v, 5 mL) at room temperature for 4 h. The resin was filtered off, and the crude peptides were precipitated from the filtrate with diethyl ether (50 mL), collected by centrifugation at 4500 rpm, and purified via RP-HPLC.

### 3 Cyclization via N → S Acyl Shift and Oxidation (Analogues **2–6**)

Linear peptides were cyclized via an N → S acyl shift in a buffer containing 0.1 M sodium phosphate (pH 5.8), 0.7 M sodium 2-mercaptoethane sulfonate, and 1.7 mM TCEP at 50 °C for 24 h. Cyclized peptides were purified by RP-HPLC, followed by oxidation using 50 mM ammonium bicarbonate (pH 8.0). The final cyclized and oxidized peptides were purified by HPLC and lyophilized. Isolated final yields (relative to the linear peptide) were as follows: **2**, 18%; **3**, 14%; **4**, 18%; **5**, 15%; **6**, 15%.

### 4 Wang Hydrazine Carbamate Resin Preparation for Analogues **7–10**

Wang resin (200 mg, 0.18 mmol, 0.91 mmol/g) was activated by treatment with *p*-nitrophenyl chloroformate (115 mg, 0.58 mmol) and *N*-methyl morpholine (64 μL) in DCM (2 mL) at 0 °C, stirred overnight at room temperature, and washed (3× DMF, 3× DCM). Hydrazinium hydroxide (63 μL, 1.26 mmol) in DMF/DCM (1.5 mL/1 mL) was then added at 0 °C. The resin was stirred overnight, filtered, and washed 3× DMF and 3× DCM.

### 5 Synthesis of Hydrazide Peptides (Analogues **7–10**)

Analogues **7–10** were synthesized manually or on an ABI433A synthesizer using hydrazine carbamate Wang resin (0.9 mmol/g, 0.05 mmol scale). Each Fmoc-amino acid (10 equiv) was preactivated with 10 equiv of HBTU, 10 equiv of HOBt, 10 equiv of DIEA, and 0.1 equiv of DMAP in DMF (1 mL) before coupling. Fmoc deprotection was achieved with 20% piperidine in DMF (1 mL, 20 min). Following cleavage with TFA/EDT/H_2_O (95:2.5:2.5, 5 mL) for 4 h, crude peptides were precipitated, centrifuged, and purified by RP-HPLC.

### 6 Cyclization via Native Chemical Ligation (Analogues **7–10**) and Oxidation

Linear peptides (1 mM) were converted to acyl azides by reaction with 10 equiv of NaNO_2_ in 6 M guanidinium chloride buffer (5 mL, pH 3–4) for 30 min on ice. MPAA (40 equiv) was added to generate the corresponding thioesters at pH 5–6, stirred for 4 h at room temperature. Disulfide-containing byproducts were reduced using TCEP (0.1 M, 30 min) and excess MPAA was removed by extraction with diethyl ether. The aqueous phase was purified by RP-HPLC. Cyclized peptides were oxidized in 50 mM ammonium bicarbonate (pH 8.0) for 24 h, then purified and lyophilized. Final isolated yields (from linear peptide) were: **7**, 8%; **8**, 6%; **9**, 9%; and **10**, 12%.

### 7 Biological Assays

The experimental procedures encompassed molecular modeling, protein–ligand docking, and a series of biochemical and cell-based assays to assess protease inhibition, calcium mobilization, and epithelial barrier function. All methodologies were performed following previously validated protocols and are further described in the Supporting Information.

## Supplementary Material











## Notes

Biological experimental methods, IC50, curves, and synthetic data, including purity (HPLC) and identity (LC-MS) (PDF)
Figure 1c (PDB)
Figure 1d (PDB)
Figure 1e (PDB)
Figure 1f (PDB)

## Abbreviations Used

AMC: 7-amino-4-methylcoumarin
HPLC: high-performance liquid chromatography
IC_50_: half maximal inhibitory concentration
KLK: kallikreins
LC-MS: liquid chromatography–mass spectrometry
LEKTI: lympho-epithelial Kazal-type-related inhibitor
PAR2: protease-activated receptor 2
SFTI: sunflower trypsin inhibitor
*SPINK*: serine protease inhibitor Kazal-type
TEER: transepithelial electrical resistance
