# Supplementary material for: LEKTI-Grafted Sunflower Trypsin Inhibitor: A Potential Therapeutic for Skin Diseases
Source: J Med Chem. 2025 Nov 11;68(22):24127–35. doi: 10.1021/acs.jmedchem.5c01912 (PMC12670400; doi:10.1021/acs.jmedchem.5c01912)
Supplement: Supplementary file 1 [file jm5c01912_si_001.pdf]

Supporting Information

LEKTI-Grafted Sunflower Trypsin Inhibitor: A Potential Therapeutic for Skin Diseases

Jeffrey Mah[a], Vignesh Jayarajan[a], Xin Huang[a], Wei-Li Di *[a], Derek Macmillan *[b]

[a]Institute of Child Health, University College London, 30 Guilford Street, London, WC1N 1EH, United Kingdom

[b]Department of Chemistry, University College London, 20 Gordon Street, London, WC1H 0AJ, United Kingdom

*Email: w.di@ucl.ac.uk

*Email: d.macmillan@ucl.ac.uk

	Content	Page
Table S1	Peptide synthesis linear sequence and yields for **2-10**	2
Figure S1.	Analogues characterisation via LC-MS and HPLC	3
Figure S2.	Optimisation for protease activity assay	12
Figure S3.	PAR2 assay establishment	13
Figure S4.	SFTI based lead 7 hydrolysis and stability assay	14
Figure S5.	IC_{50} curves for synthesized analogues **2-6**	15
	Methods for biological experiment	16
	References	21

Table S1. Peptide synthesis linear sequence and yields for 2-10

Linear peptide to cyclic precursor:	Synthesis sequence	Theoretical yield (mg)	Actual yield (mg)	% Yield
**2**	H-CWPDGRCTRSIPPHC-OH	99.9	47.5	47
**3**	H-CWPNGFCTRENPPHC-OH	101.6	59.9	59
**4**	H-CWPNGFCHREYPPHC-OH	104.0	56.8	54
**5**	H-CWPNGFCTRSNPPHC-OH	99.5	51.7	51
**6**	H-CWPNGFCHRSYPPHC-OH	103.7	44.7	43
**7**	H-CTRSNPPECWPNGF-$NHNH_2$	79.4	28.3	35
**8**	H-CHRSYPPECWPNGF- $NHNH_2$	84.7	22.6	26
**9**	H-CTRSSPPECWPNGS- $NHNH_2$	75.1	26.2	34
**10**	H-CTRSSPPECWPNGY- $NHNH_2$	78.9	31.8	40

Figure S1. Analogues characterisation via LC-MS and HPLC

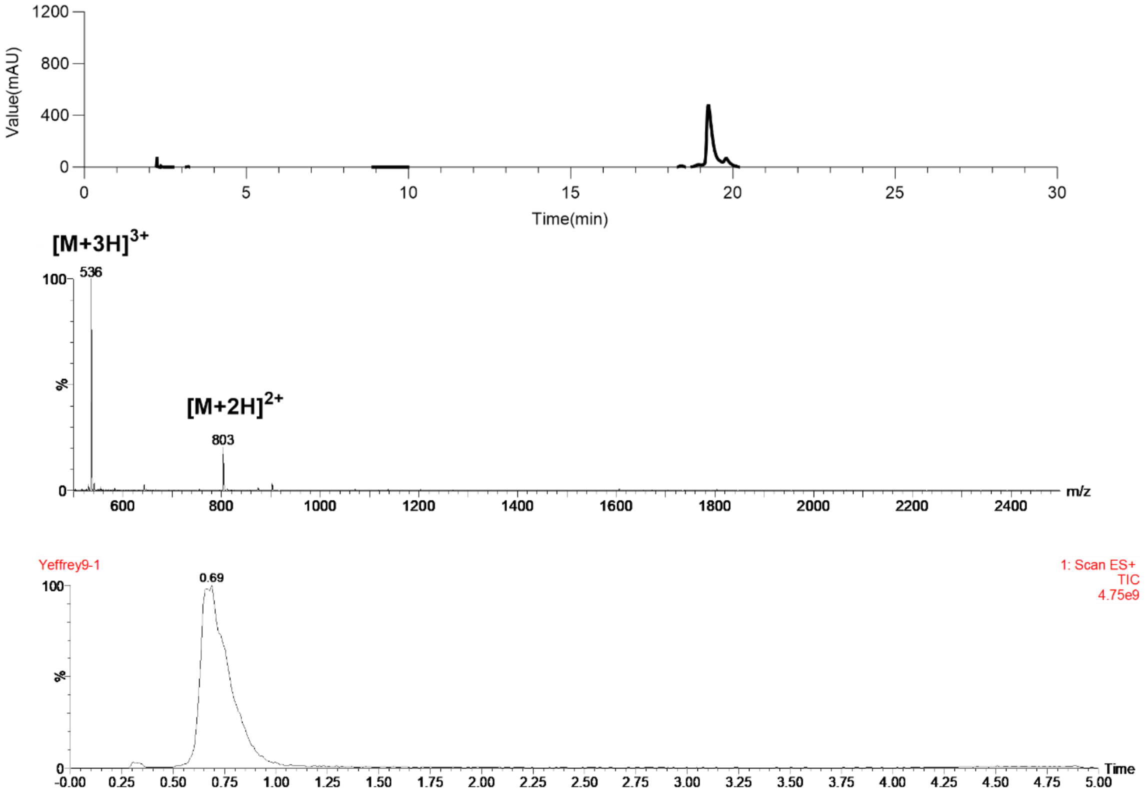

a) *SFTI-derived **2**: Purified final cyclised form with disulphide bridge formation analysed using LC-MS (Observed: 1605 Da; Calculated: 1605 Da). Analytical HPLC at 280nm demonstrated purity over 95% pure with single peak corresponds to the peptide.*

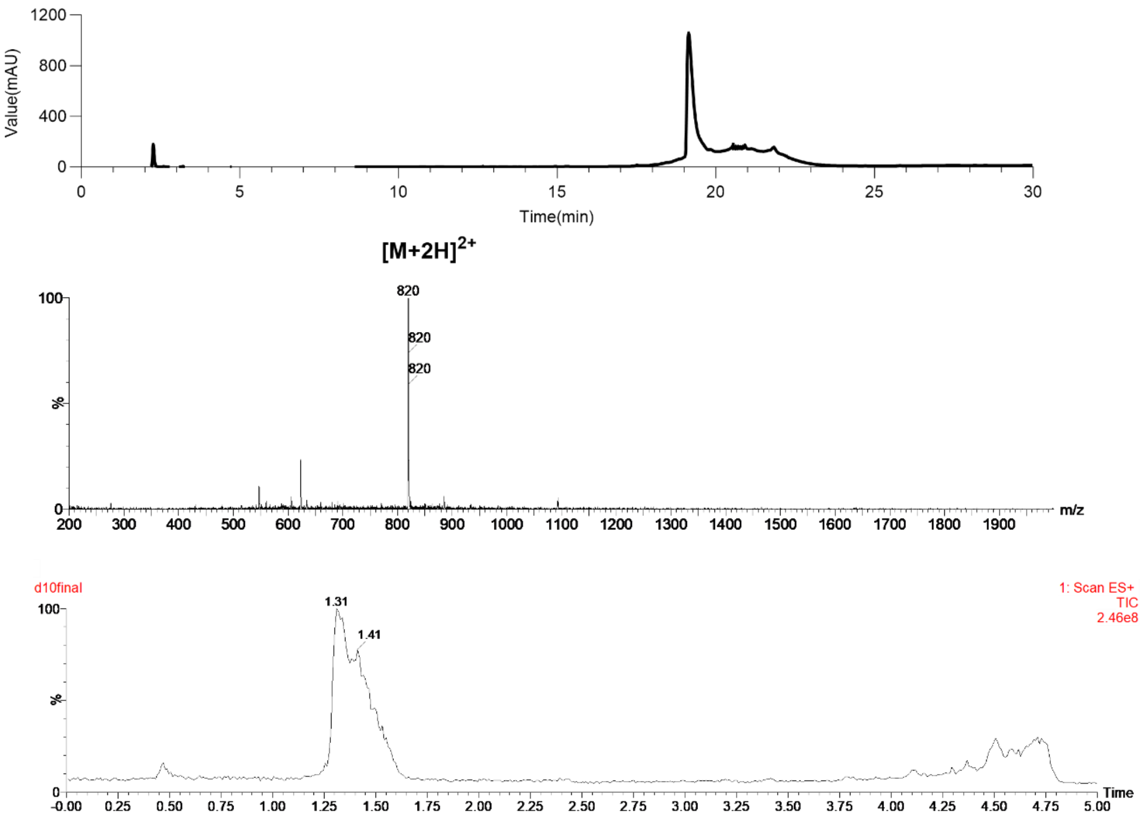

b) *SFTI-derived **3**: Purified final cyclised form with disulphide bridge formation analysed using LC-MS (Observed: 1638 Da; Calculated: 1638 Da). Analytical HPLC at 280nm demonstrated purity over 95% pure with main single peak corresponds to the peptide.*

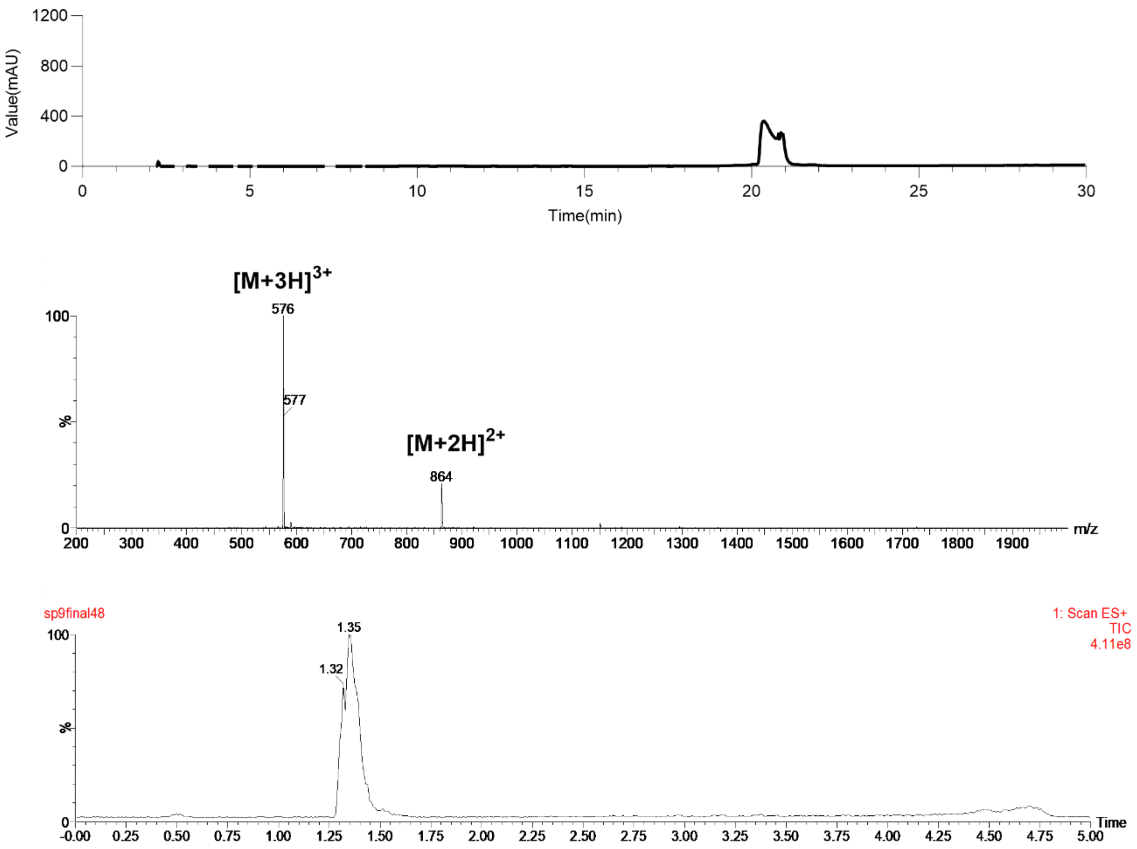

c) *SFTI-derived **4**: Purified final cyclised form with disulphide bridge formation analysed using LC-MS (Observed: 1725 Da; Calculated: 1725 Da). Analytical HPLC at 280nm demonstrated purity over 95% pure with main single peak corresponds to the peptide.*

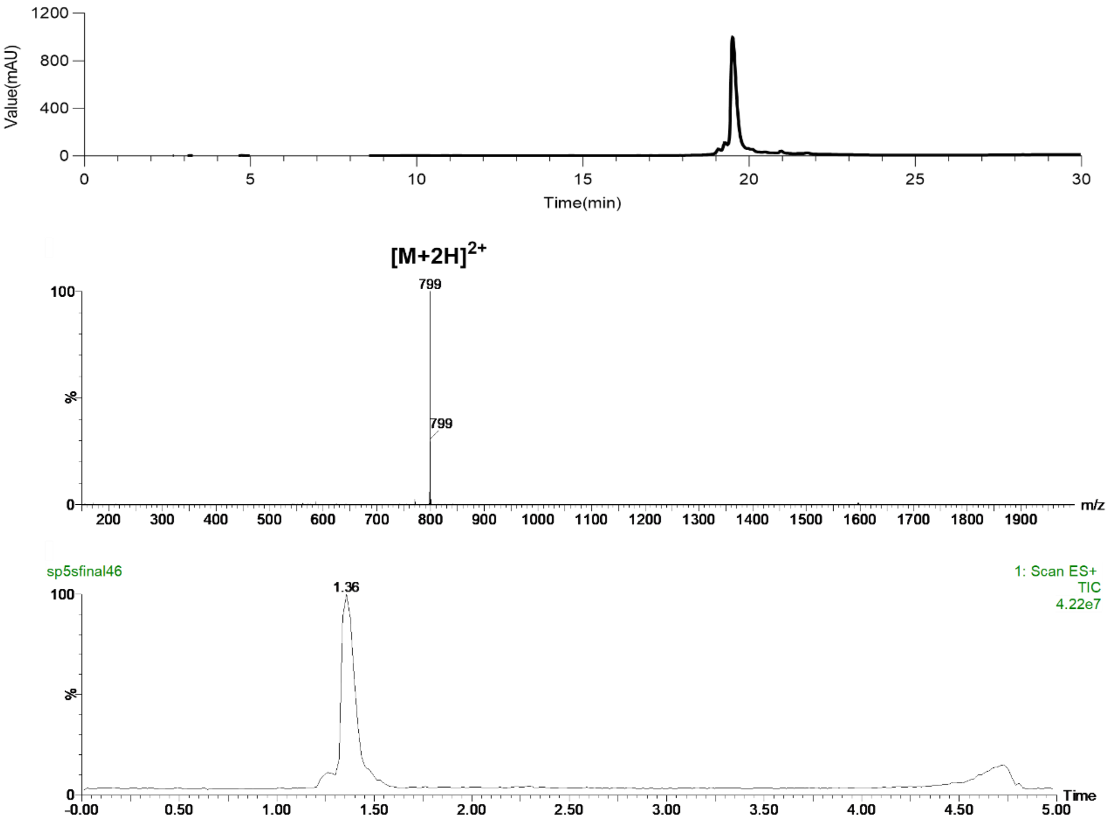

d) *SFTI-derived **5**: Purified final cyclised form with disulphide bridge formation analysed using LC-MS (Observed: 1596 Da; Calculated: 1596 Da). Analytical HPLC at 280nm demonstrated purity over 95% pure with main single peak corresponds to the peptide.*

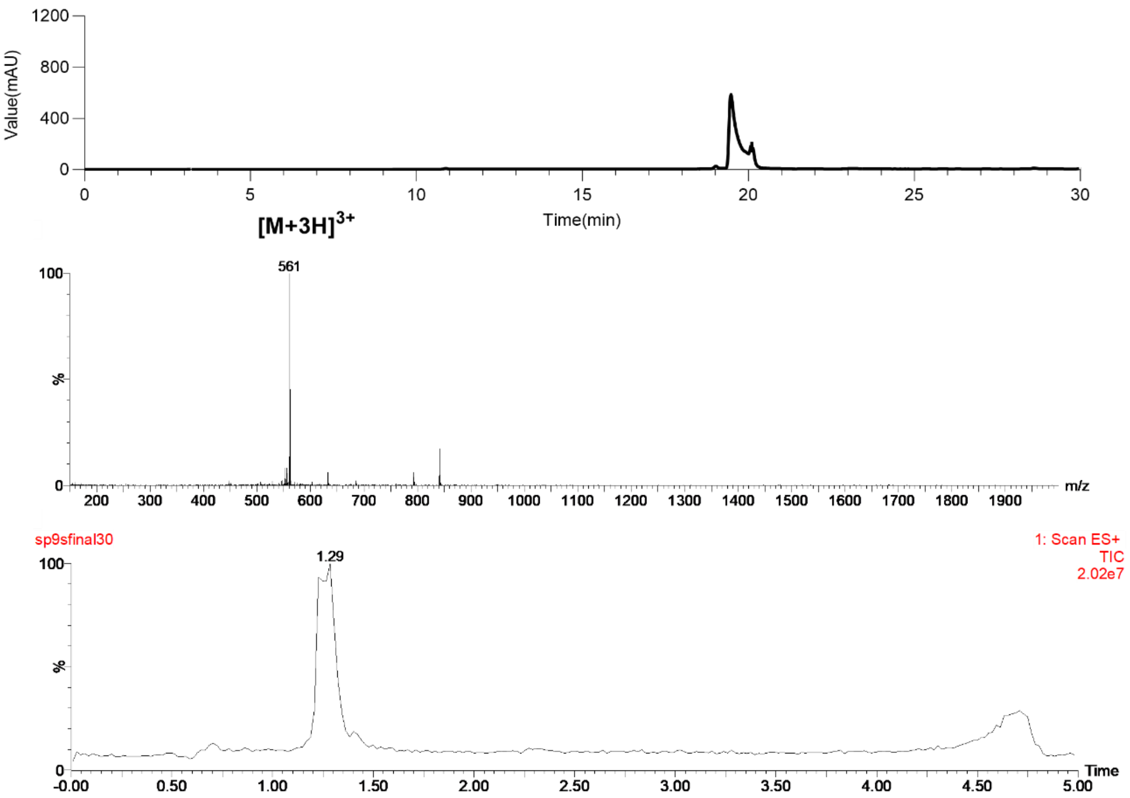

*SFTI-derived **6**: Purified final cyclised form with disulphide bridge formation analysed using LC-MS (Observed: 1680 Da; Calculated: 1681 Da). Analytical HPLC at 280nm demonstrated purity over 95% pure with main single peak corresponds to the peptide.*

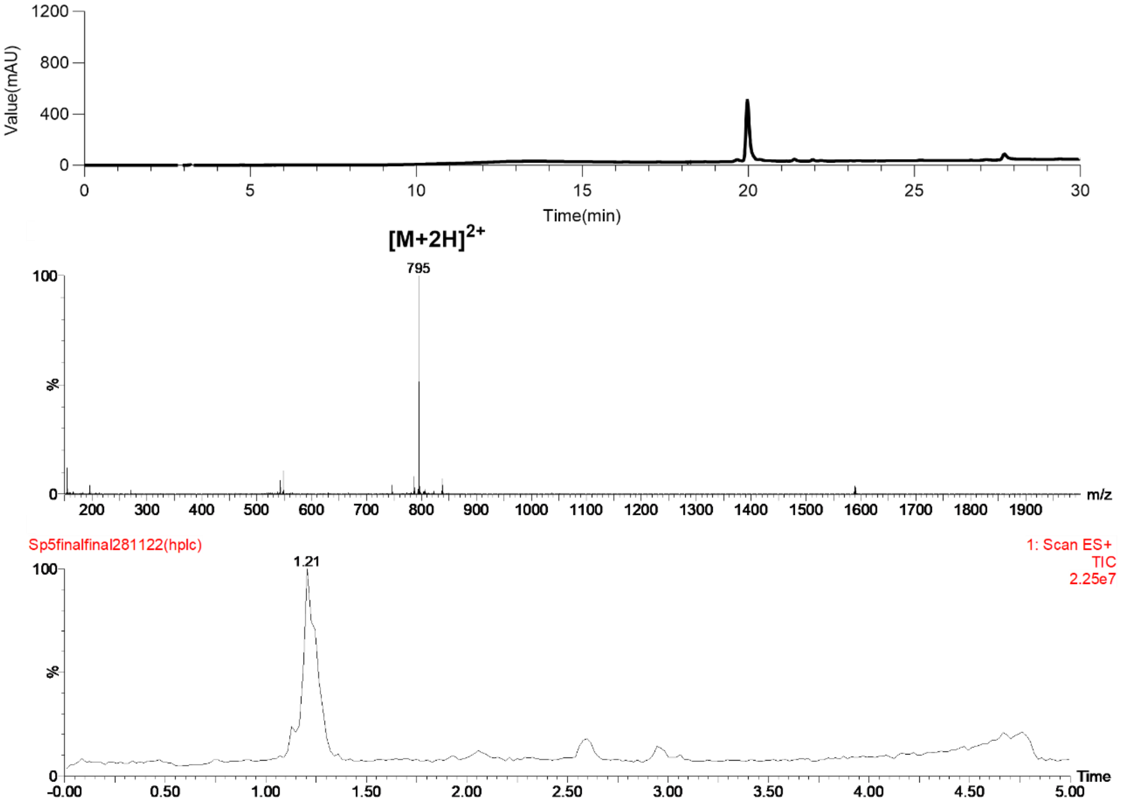

e) SFTI-derived ***7****: Purified final cyclised form with disulphide bridge formation analysed using LC-MS (Observed: 1588 Da; Calculated: 1587.76 Da). Analytical HPLC at 280nm demonstrated purity over 95% pure with main single peak corresponds to the peptide.*

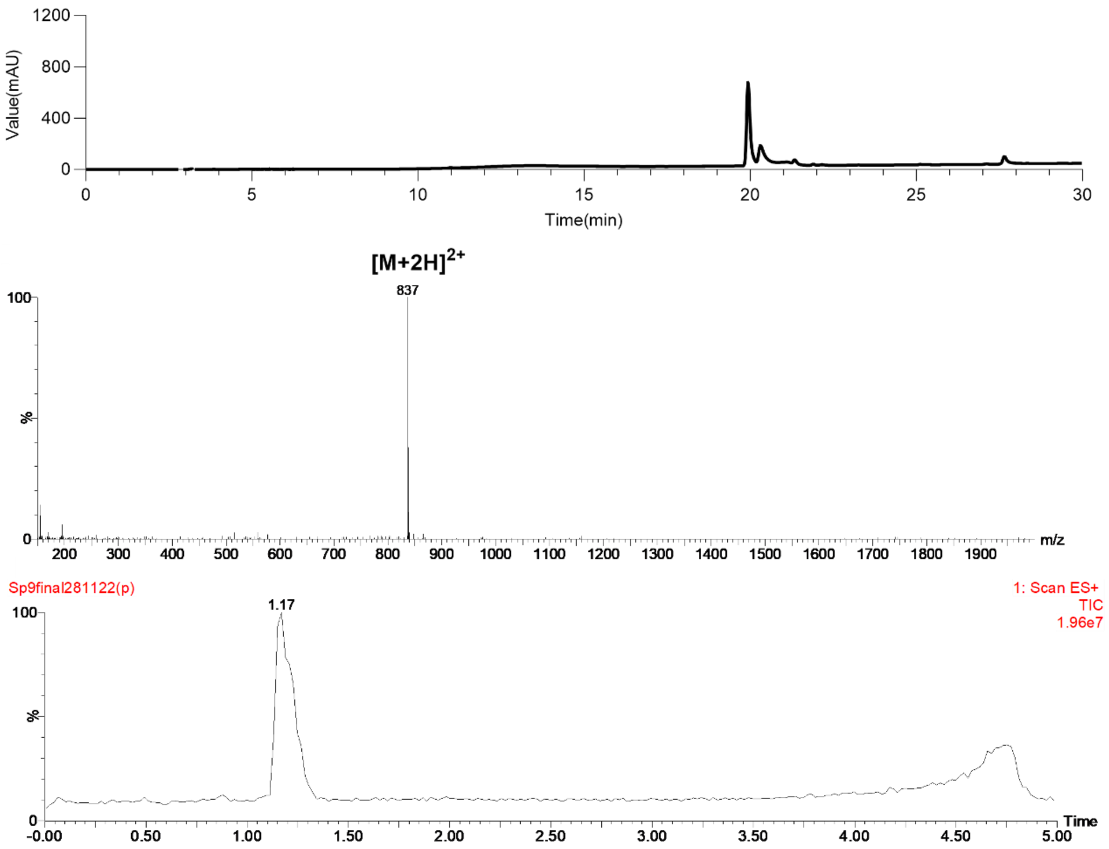

f) *SFTI-derived **8**: Purified final cyclised form with disulphide bridge formation analysed using LC-MS (Observed: 1672 Da; Calculated: 1673 Da). Analytical HPLC at 280nm demonstrated purity over 95% pure with main single peak corresponds to the peptide.*

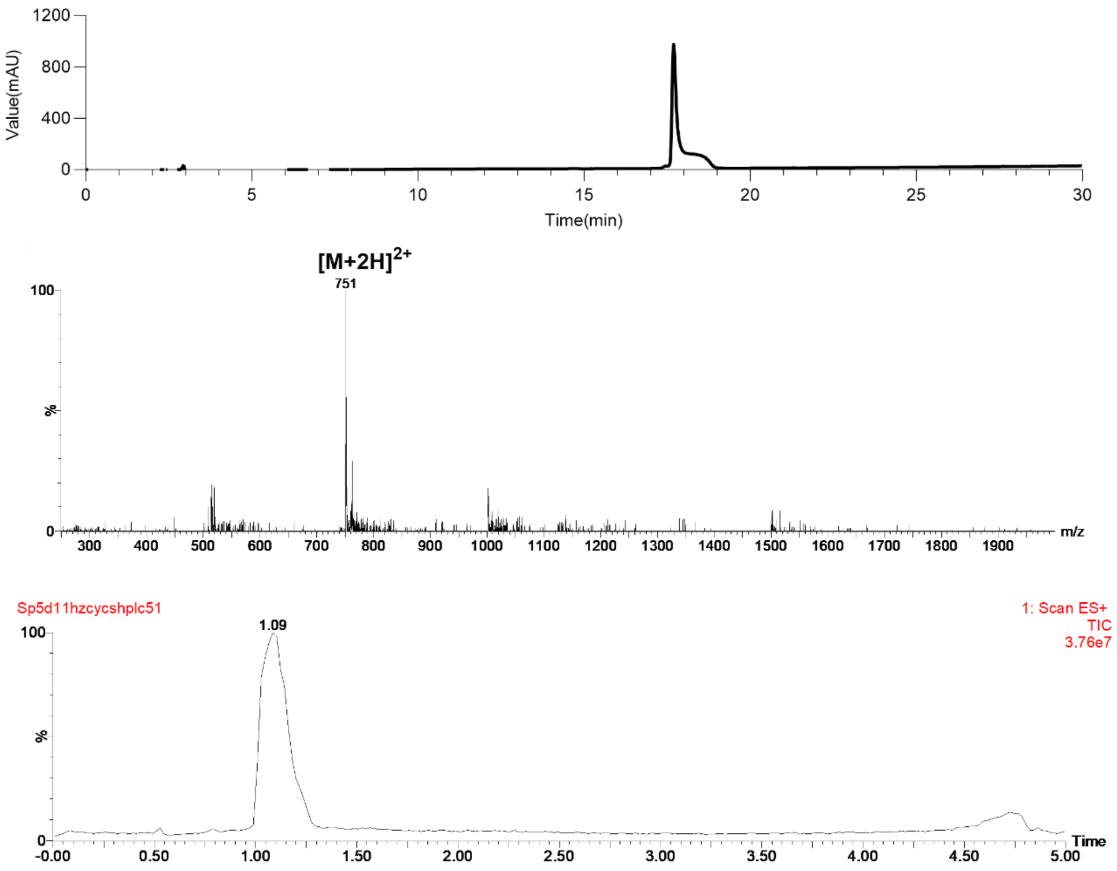

*g) SFTI-derived **9**: Purified final cyclised form with disulphide bridge formation analysed using LC-MS (Observed: 1500 Da; Calculated: 1500.62 Da). Analytical HPLC at 280nm demonstrated purity over 95% pure with main single peak corresponds to the peptide.*

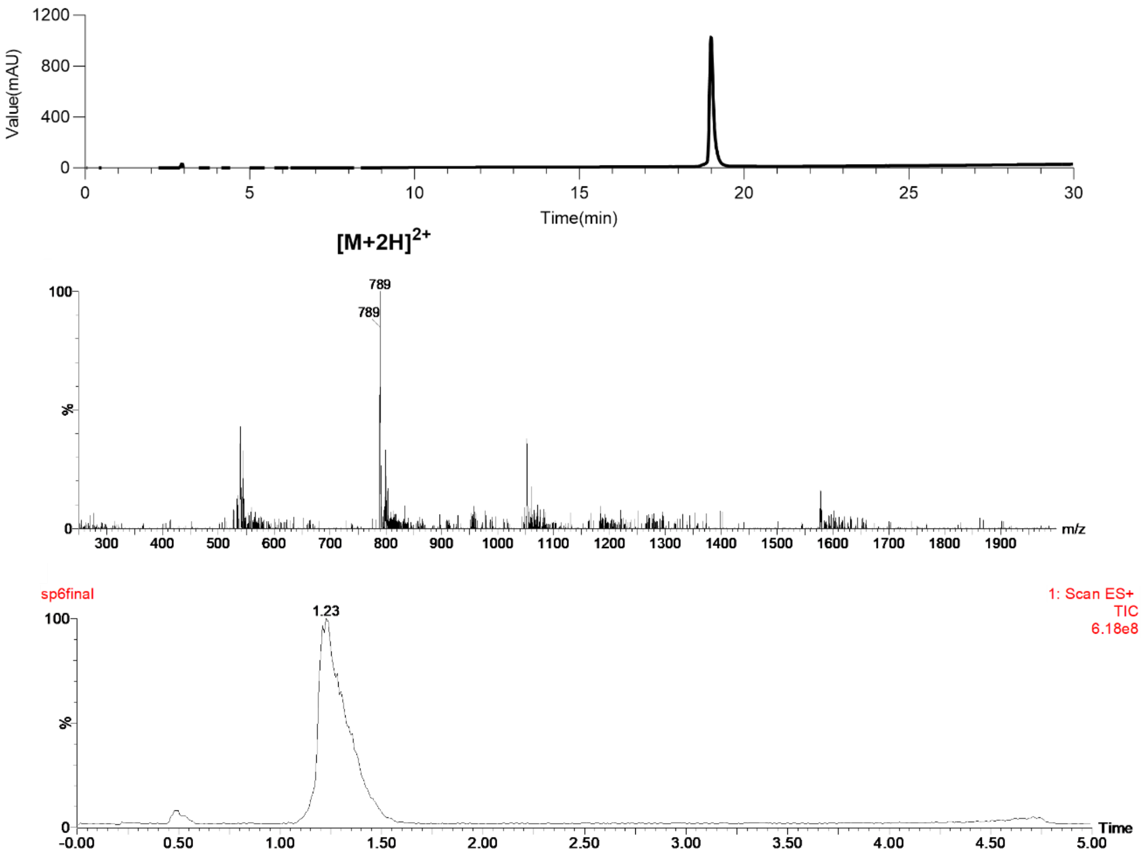

h) *SFTI-derived **10**: Purified final cyclised form with disulphide bridge formation analysed using LC-MS (Observed: 1576 Da; Calculated: 1576.74 Da). Analytical HPLC at 280nm demonstrated purity over 95% pure with main single peak corresponds to the peptide.*

Figure S2. Optimisation for protease activity assay

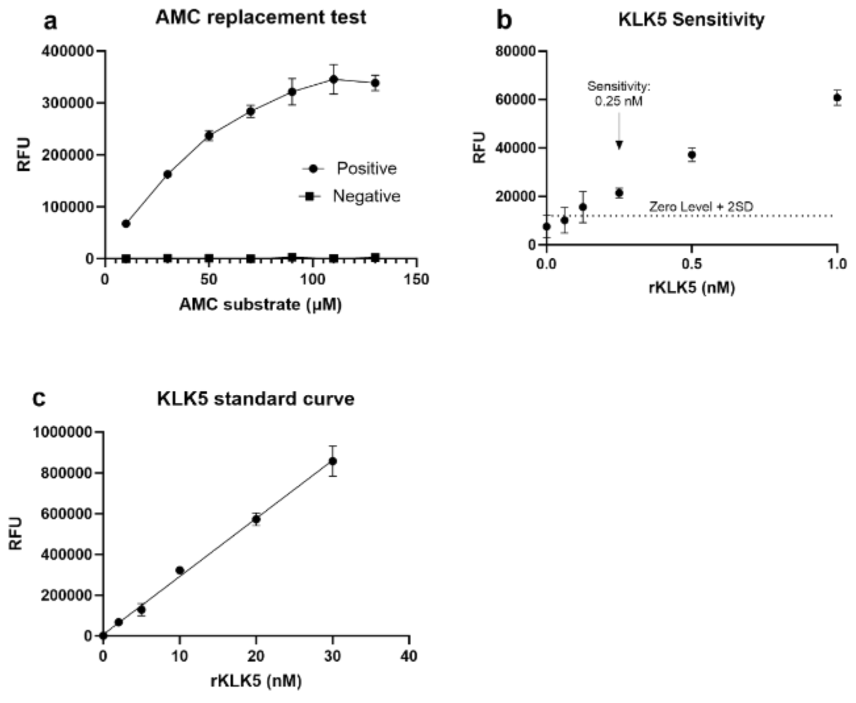

Optimization of the assay for reliable detection of KLK5 activity using AMC assay.

(a) Various concentration of AMC substrates was reacted with 10 nM rKLK5, with maximum fluorescence intensity at 110 µM. (b) The sensitivity of AMC assay was determined as 0.25 nM for KLK5, where the concentrations minus 2SD did not overlap with the zero concentration plus 2SD (n = 10 for each concentration). (c) Linearity of rKLK5 standard curve (n=3).

Figure S3. PAR2 assay establishment

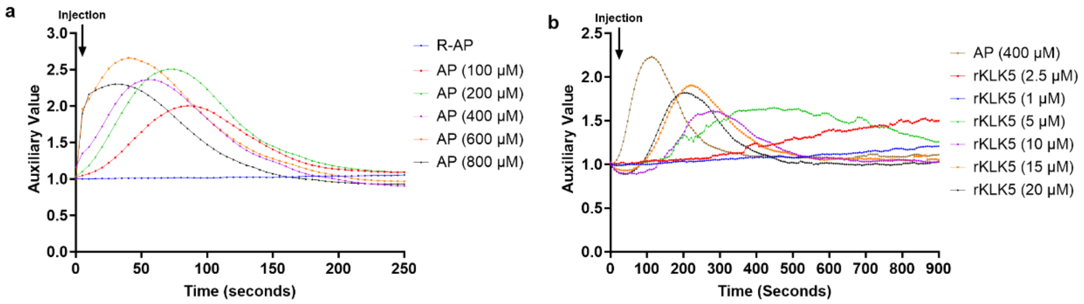

KLK5-Induced Intracellular Calcium Mobilization optimisation and controls for PAR2

(a) PAR2 agonist and agonist control were added and tested to ensure the reliability of assay, presence of PAR-2 receptors, and diluent did not cause a signal. (b) Change in relative fluorescence unit (ΔRFU) for 10 minutes after injections of mixture of 10 μM (833.3 nM f/c) KLK5 and different concentrations of lead ***7****. Lead* ***7*** *achieved inhibitor-to-enzyme ratio of 1:1. Data is smoothed and normalized to auxiliary units using initial background reading. Concentrations indicated are final concentration in well and all reagents are diluted using 1xPBS pH 8.0.*

Figure S4. SFTI based lead 7 hydrolysis and stability assay

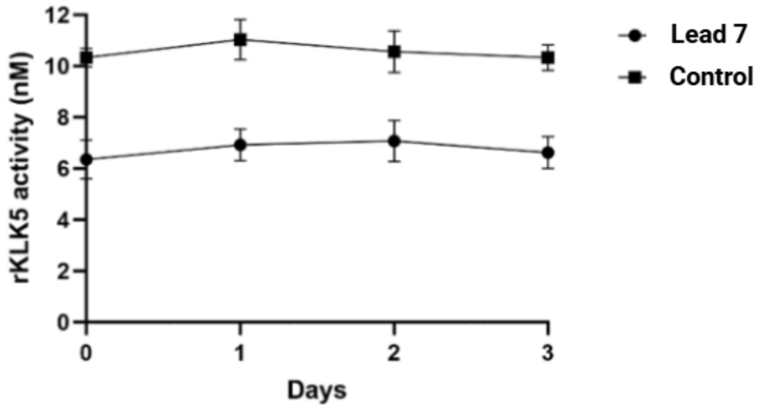

Stability of lead 7 in heat inactivated FBS medium

A concentration of 10 nM of lead ***7*** *was incubated in heat-inactivated fetal bovine serum (FBS) medium without cells for a period of three days. Following incubation, lead* ***7****'s residual inhibitory activity against recombinant kallikrein-related peptidase 5 (rKLK5) was evaluated using an AMC (7-amino-4-methylcoumarin) fluorogenic substrate assay. For comparison, control samples consisting of medium without lead* ***7*** *were prepared and used as the negative control (control). Experiment showed no change in inhibitory activity against rKLK5 for lead* ***7*** *for over 3 days, indicating its stable and not prone to hydrolysis when in culturing medium. Error bars represent mean rKLK5 activity with one standard deviation from three independent experiments (n = 3).*

Figure S5. IC_{50} curves for analogues 2, 3, 4, 5, & 6

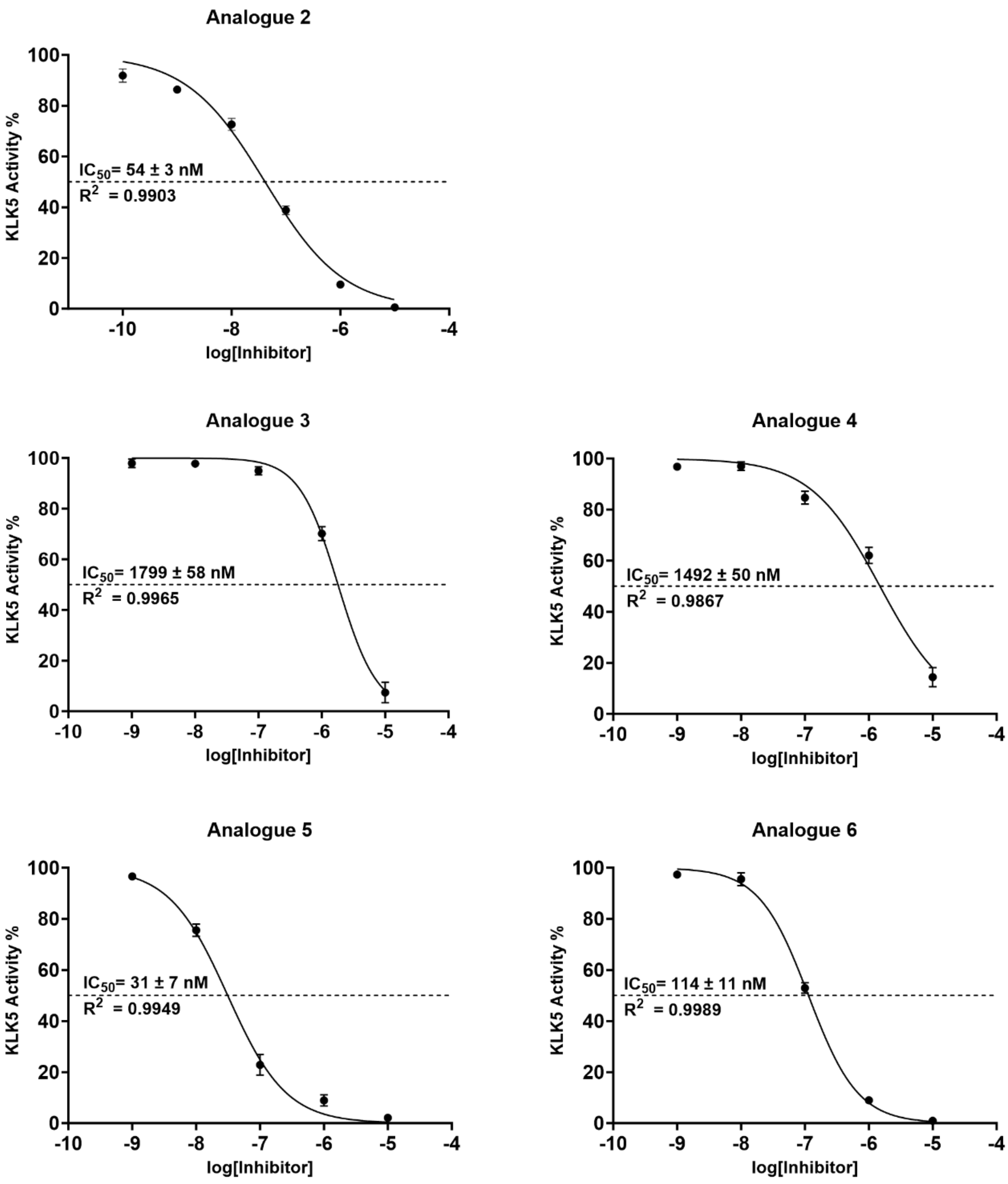

Normalized dose–response curves showing KLK5 inhibition by ***2, 3, 4, 5,*** *&* ***6****. Data were fitted using nonlinear four-parameter logistic regression. IC_{50} values were determined as 54 ± 3 nM for Analogue 6, 114 ± 11 nM for S-9012, and 31 ± 7 nM for S-5102. Data points represent the mean of three independent experiments, and error bars indicate standard deviation.*

Methods for biological experiment

Computer modelling

SFTI analogues were created by individual amino acid substitutions in PYMOL (Python Molecular Graphics System) using the existing solution NMR structure of native SFTI-1 (1JBL) found in the RCSB (Research Collaboratory for Structural Bioinformatics) database. Residues of **2** were mutated as follows: L5R, I10H, and F12W with conserved SFTI-1 backbone structure. Other SFTI based analogues were further modified using PYMOL. Currently, there are no SFTI-1 and Kallikrein 5 co-crystal structures found in the RCSB database. Superimposing the existing crystal structure of SFTI-1 and Trypsin complex (4XOJ) with Kallikrein 5 crystal structure (2PSX) was performed with PYMOL. Ab-initio docking between KLK5 and **2** were performed with HADDOCK 2.4 [1, 2], with binding active site parameter set to Arg2, Cys3, Thr4, Arg5, Ser6 for **2** and Ser195, His57, and Asp102 for KLK5 catalytic triad. PRODIGY [3-5], set at 25 °C, was used to predict dissociation constant and binding interactions of docked clusters. Structural visualization and alignment were performed with PYMOL [6], and comparative sequence analysis was performed with MEGA11[7].

Protease activity assay

A fluorescence spectrometer using SpectraMax® i3x plate reader (Molecular Devices, United States) was used to measure the relative fluorescence unit (RFU) on a 96-well black microplate (Grenier Bio-one, cat no. 655074). The wavelengths were set to 360/460 nm (excitation/emission) for the substrate Boc-VPR-AMC (Bachem AG, cat no. 4003460) and 320/405nm for the substrate Mca-RPKPVE-Nval-WRK(Dnp)-NH_2 Fluorogenic MMP Substrate (R&D Systems Biotechne, cat no.ES002). The Boc-VPR-AMC stock solution was prepared by dissolving the solid form in dimethyl sulfoxide to 100 mM. Mca-RPKPVE-Nval-WRK(Dnp)-NH_2 fluorogenic MMP Substrate is supplied as stock solution at 10.0 mg/mL in dimethyl sulfoxide. Boc-VPR-AMC was used as a substrate for KLK5, 8 and 14. Mca-RPKPVE-Nval-WRK(Dnp)-NH_2 was used as a substrate for KLK7. The ligand-only solution serves as a negative control (minimum RFU, 100% inhibition), and enzyme-only was served as positive (maximum RFU, 0% inhibition). The activities of proteases were measured and readout against the standard curve made in serial dilutions of tested recombinant KLKs, ranging from 0nM to 60nM. Each assay was done in triplicates and repeated at least for three times

Assessment of analogue mediated inhibition of KLK5, KLK7, KLK8, and KLK14 protease activity

Recombinant KLK5 (R&D Systems, cat no. 1108-SE) was diluted to a final concentration of 10 nM in 1X PBS (pH 8.0). KLK5 was pre-incubated on ice for 1 hour with serial dilutions of SFTI-derived analogues to assess inhibitory activity. After incubation, 20 µL of each mixture was transferred to wells of a 96-well microplate in triplicates. The enzymatic reaction was initiated by adding 100 µL of Boc-VPR-AMC substrate (final concentration 100 µM) to each well. Fluorescence (RFU) was measured using a SpectraMax® i3x plate reader with chamber temperature maintained at 37°C. Readings were started at 0 minutes (baseline) and another reading at 15 minutes.

Recombinant KLK7 (R&D Systems, cat no. 2624-SE) and KLK14 (R&D Systems, cat no. 2664-SE) were activated by adding 1 mM $CaCl_2$ 0.05% v/v Brij-35 (Sigma, cat no. 8019620250), and 20 nM thermolysin (Sigma, cat no. P1512). The mixtures were incubated at 37°C for 1 hour. Reactions were stopped by adding EDTA to a final concentration of 50 mM (Sigma, cat no. EDS-100G) KLK8 (catalytic domain only; R&D Systems, cat no. 2025-SE-010) was activated separately with 11 nM lysyl endopeptidase (Santa Cruz Biotechnology, cat no. sc-360250C) and incubated at 37°C for 30 minutes. Recombinant KLK7, KLK8, and KLK14 were each diluted in 1X PBS (pH 8.0) to a final concentration of 100 nM. KLK activity assays for KLK8 and KLK14 were carried out at 25°C, with fluorescence readings recorded 15 minutes after substrate addition. In contrast, KLK7 assays were performed at 37°C with an extended incubation of 20 hours, due to its lower enzymatic activity, which necessitated a longer period to produce a measurable fluorescence signal.

IC_{50} curve was fitted and analysed using Prism 10. All tested compounds and samples were reconstituted in 1x PBS (pH 8.0). The percentage of inhibition was normalized using the following equation:

$$\%\text{ of Inhibition} = \left(1 - \frac{(\text{Sample RFU} - \text{Min RFU})}{(\text{Max RFU} - \text{Min RFU})}\right) \times 100\%$$

For Ki calculation, Km values for KLK5 and its substrate Boc-VPR-AMC were previously reported in the literature [8, 9], allowing the conversion from IC_{50} to K_i using the Cheng Prusoff equation[10] $K_i = IC_{50} / (1 + [S]/K_m)$ for single-site competitive binding.

Cell lines and tissue cultures

Keratinocytes isolated from the skin in a patient with genetic skin disease Netherton Syndrome (NS19) and in healthy donors (C42, C46, P8) were used in the study. Written consent was obtained from the patient and health donors' guardians, and ethical approval was granted by the local research ethics committee (REC reference: 12/LO/1522). These cells were immortalised by E6/E7 gene in HPV [11] and the immortalised cell lines were used in this study

Keratinocyte cell lines were cultured in RM+ medium, composed of equal parts of DMEM (Thermo scientific, cat no. 31966047) and DMEM/Ham F12 (Thermo Scientific cat no. 11320033), supplemented with 10% fetal bovine serum (FBS), 10% penicillin/streptomycin (Pen/Strep), and another growth factors, including 10 ng/mL human epidermal growth factor, 0.4 µg/mL hydrocortisone, 5 µg/mL transferrin, 5 µg/mL insulin, 2×10^{-11} M liothyronine sodium salt, and 1×10^{-10} M cholera toxin. To support keratinocyte growth, irradiated murine 3T3 cells were co-cultured with keratinocytes. Cells were incubated at 37°C with 10% CO_2[12].

Intracellular Calcium Mobilization Assay

The immortalized keratinocyte cell line (N/TERT)[13, 14] were used for the assay. N/TERT cells were harvested and resuspended in fresh DMEM RM+ culture medium 24 hours prior to the assay. The cells were counted, and 100 µL of the suspension, at a density of 600,000 cells/mL, was seeded into each well of a 96-well microplate. The plate was incubated at 37°C with 10% CO_2 for 24 hours before the assay. On the day of the assay, the FLUOFORTE® Dye-Loading Solution from the FLUOFORTE® Calcium Assay Kit (ENZO Biochem, cat no. ENZ-51017) was applied to the cells to enable calcium signal detection. PAR-2 functionality was confirmed by injecting 20 µL of a 400 µM PAR-2 agonist peptide (AP, SLIGRL-NH_2, Abcam, cat no. ab120176) along with recombinant KLK5, followed by the measurement of RFU changes of intracellular

calcium signalling. For negative controls, 20 μL of a 400 μM reverse sequence PAR-2 agonist peptide (R-AP, LRGILS-NH_2, Bio-techne, cat no.3394/1) and 1x PBS (pH 8.0) were used. The inhibition of analogues on KLK5 were evaluated by incubating serially diluted lead **7** with 10 μM recombinant KLK5 in 1X PBS, mixed with a 20 μL aliquot of the AP and then injected into cells plated on the day before. fluorescence changes (RFU) in each well were recorded immediately starting from time 0 and lasted for 10 minutes at 5-second intervals at 37°C. Fluorescence excitation was set at 485 nm, and emission was recorded at 520 nm using SpectraMax® i3x plate reader.

KLK5 protease activity assay for culture medium

Keratinocytes were seeded in 6 well plate with the density of 0.2 × 10^6 cells per well and cultured in RM+ medium until they reached confluency. The culture media were changed to FBS free-RM+ media, and cells were continuously cultured. 48 hours after the medium changing, the culture media were harvested from the cell culture plates and centrifuged to remove dead cells. A volume of 180 μL of the supernatants was transferred to 1.5 mL Eppendorf tubes, followed by the addition of 20 μL of analogues in 1x PBS at different concentrations. The samples in which 20 μL of 1x PBS instead of the lead **7** were added were as untreated controls. These mixtures were incubated for one hours at 37 degrees Celsius and then processed for precipitation to concentrate the samples using ProteoExtract® Protein Precipitation Kit (Millipore, cat no. 539180). The precipitates were resuspended in 20 μL of 1X PBS (pH 8.0) for protease activity assessment using protease activity assay. 10uL of suspension from each sample was added in 96 well black plate. The enzymatic reaction was initiated by adding 100 μL of Boc-VPR-AMC substrate (final concentration 100 μM) to each well and RFU values in samples were measured. Protease activities were expressed as nmol/g by first converting RFU readings to KLK5 equivalent concentration activity using a KLK5 standard curve, followed by normalization to total protein content quantified in μg/μL.

Transepithelial Electrical Resistance (TEER) assay

Keratinocyte cell lines NS19 (derived from a Netherton syndrome patient), and C42, C46, and P8 (from healthy donors) were seeded onto 6-well cell culture inserts. (THINCERT®, Greiner Bio-One, cat no. 657610) at a density of 0.5×10^6 cells per well. The inserts were placed into standard 6-well plates and cells were cultured in the RM+ medium containing lead **7** at a final concentration of 1 μM. Negative control wells without addition of SFTI-based analogues were run in parallel.

Transepithelial electrical resistance (TEER) values in ohms (Ω) were measured using the EVOM™ Manual Resistance Meter (World Precision Instruments, cat no. EVM-MT-03-02). Background resistance was first recorded using inserts containing only medium (without cells). TEER measurements were then performed in three times for each well to get mean values.

Statistical analysis

One-way ANOVA was used for KLK5 activity study. Where appropriate, pairwise comparisons between treatments were conducted using Tukey's post hoc test with adjustment for multiple testing. TEER study data was analysed using the Wilcoxon signed-rank test.

A significance threshold of $p < 0.05$ was applied throughout all analyses. Statistical analysis and visualisation were performed using GraphPad Prism 10.

References

1. G. C. P. van Zundert, J. P. G. L. M. Rodrigues, M. Trellet, C. Schmitz, P. L. Kastritis, E. Karaca, A. S. J. Melquiond, M. van Dijk, S. J. de Vries and A. M. J. J. Bonvin, *J Mol Biol*, 2016, **428**, 720-725.
2. C. Dominguez, R. Boelens and A. M. J. J. Bonvin, *J Am Chem Soc*, 2003, **125**, 1731-1737.
3. L. C. Xue, J. P. Rodrigues, P. L. Kastritis, A. M. Bonvin and A. Vangone, *Bioinformatics*, 2016, **32**, 3676-3678.
4. A. Vangone and A. M. J. J. Bonvin, *Elife*, 2015, **4**.
5. R. V. Honorato, P. I. Koukos, B. Jiménez-García, A. Tsaregorodtsev, M. Verlato, A. Giachetti, A. Rosato and A. M. J. J. Bonvin, *Front Mol Biosci*, 2021, **8**.
6. Schrodinger, LLC, unpublished work.
7. K. Tamura, G. Stecher and S. Kumar, *Mol Biol Evol*, 2021, **38**, 3022-3027.
8. C. Jendrny and A. G. Beck-Sickinger, *Chembiochem*, 2016, **17**, 719-726.
9. I. P. Michael, G. Sotiropoulou, G. Pampalakis, A. Magklara, M. Ghosh, G. Wasney and E. P. Diamandis, *J Biol Chem*, 2005, **280**, 14628-14635.
10. Y. Cheng and W. H. Prusoff, *Biochem Pharmacol*, 1973, **22**, 3099-3108.
11. K. Munger, W. C. Phelps, V. Bubb, P. M. Howley and R. Schlegel, *J Virol*, 1989, **63**, 4417-4421.
12. Y. N. Zhu, J. Underwood, D. Macmillan, L. Shariff, R. O'Shaughnessy, J. I. Harper, C. Pickard, P. S. Friedmann, E. Healy and W. L. Di, *J Allergy Clin Immun*, 2017, **140**, 1310-+.
13. J. P. H. Smits, H. Niehues, G. Rikken, I. van Vlijmen-Willems, G. van de Zande, P. Zeeuwen, J. Schalkwijk and E. H. van den Bogaard, *Sci Rep*, 2017, **7**, 11838.
14. M. A. Dickson, W. C. Hahn, Y. Ino, V. Ronfard, J. Y. Wu, R. A. Weinberg, D. N. Louis, F. P. Li and J. G. Rheinwald, *Mol Cell Biol*, 2000, **20**, 1436-1447.
